# Cartilage Extracellular Matrix Scaffold With Kartogenin-Encapsulated PLGA Microspheres for Cartilage Regeneration

**DOI:** 10.3389/fbioe.2020.600103

**Published:** 2020-12-09

**Authors:** Yanhong Zhao, Xige Zhao, Rui Zhang, Ying Huang, Yunjie Li, Minhui Shan, Xintong Zhong, Yi Xing, Min Wang, Yang Zhang, Yanmei Zhao

**Affiliations:** ^1^Stomatological Hospital of Tianjin Medical University, Tianjin, China; ^2^Tianjin Medical University, Tianjin, China; ^3^Tianjin Hospital, Tianjin, China; ^4^Institute of Disaster Medicine, Tianjin University, Tianjin, China

**Keywords:** cartilage tissue engineering, decellularized cartilage extracellular matrix, poly(lactic-co-glycolic acid), composite scaffold, kartogenin

## Abstract

Repair of articular cartilage defects is a challenging aspect of clinical treatment. Kartogenin (KGN), a small molecular compound, can induce the differentiation of bone marrow-derived mesenchymal stem cells (BMSCs) into chondrocytes. Here, we constructed a scaffold based on chondrocyte extracellular matrix (CECM) and poly(lactic-co-glycolic acid) (PLGA) microspheres (MP), which can slowly release KGN, thus enhancing its efficiency. Cell adhesion, live/dead staining, and CCK-8 results indicated that the PLGA(KGN)/CECM scaffold exhibited good biocompatibility. Histological staining and quantitative analysis demonstrated the ability of the PLGA(KGN)/CECM composite scaffold to promote the differentiation of BMSCs. Macroscopic observations, histological tests, and specific marker analysis showed that the regenerated tissues possessed characteristics similar to those of normal hyaline cartilage in a rabbit model. Use of the PLGA(KGN)/CECM scaffold may mimic the regenerative microenvironment, thereby promoting chondrogenic differentiation of BMSCs *in vitro* and *in vivo*. Therefore, this innovative composite scaffold may represent a promising approach for acellular cartilage tissue engineering.

## Introduction

Because of its avascular, alymphatic, and aneural properties, articular cartilage has poor spontaneous repair and regeneration capabilities (Malfait, 2016). The widespread articular cartilage injury caused by trauma, infection, osteoarthritis, tumors, and other diseases is a challenging aspect of clinical treatment (Huang et al., 2016). Traditional treatment methods (e.g., mosaicplasty, autologous chondrocyte transplantation, and microfracture surgery) have a number of limitations, including poor repair effect and additional trauma (Loeser et al., 2016). In the past decade, tissue engineering, especially cell-free tissue engineering, which involves scaffold implantation and microfracture creation to promote the recruitment of endogenous cells has been studied to overcome these limitations (Zhen et al., 2013; Trounson and McDonald, 2015).

Stimulating factors are among the most basic elements for cell-free tissue engineering. Previous studies have focused on cartilage stimulation methods that support the capacity for self-repair. Signaling molecules, such as transforming growth factor beta-3 (TGF-β3), have been widely used as chondrogenic modulators (Mi et al., 2003; van der Kraan and van den Berg, 2007; Cals et al., 2012; Almeida et al., 2014). Kartogenin (KGN), a non-toxic and stable small molecule first described by Johnson et al. (2012) is sufficiently small to avoid immune responses; it demonstrates good stability, cost-effectiveness, and ease of storage and transportation, compared to signaling molecules (e.g., TGF-β3). During mesenchymal stem cell (MSC) differentiation, KGN frees CBFβ, which binds to the transcription factor RUNX1. The resultant CBFβ-RUNX1 complex plays a critical role in the activation of cartilage matrix transcription; therefore, KGN has great potential for use in cartilage regeneration (Wu et al., 2014). Furthermore, KGN has protective effects with respect to existing cartilage tissue and reduces the decomposition of chondrogenic proteins; thus, it slows the progression of cartilage injury (Cai et al., 2019). Although KGN has been investigated as a cartilage-promoting molecule, two common models of KGN application remain problematic: (1) direct injection into the joint cavity, (Xu et al., 2015; Kwon et al., 2018) which is ineffective because most KGN is absorbed by the circulatory system and repeated injections increase both pain and risk of infection; (2) combination of KGN with drug delivery system: such as hydrogel (Wang S.J. et al., 2019), platelet-rich plasma (PRP)(Liu et al., 2019), nanoparticles, or microspheres (Kang et al., 2014). Although several drug delivery systems have been used to achieve sustained release of KGN, the potential application of KGN in cartilage tissue engineering is seldom investigated, and additional mesenchymal stem cells implantation are needed (Li et al., 2016; Wang J. et al., 2019). Therefore, methods for application of KGN would need to be further studied. Previous studies have shown that scaffolds loaded with drug-encapsulated microspheres can achieve sustained drug release and ensure that cells within the scaffold maintain high levels of activity (Gupta et al., 2017; Zhang et al., 2017). Among the most attractive polymer candidates, poly(lactic-co-glycolic acid) (PLGA)—a typical compound used in fabrication of biodegradable microspheres or nanoparticles, which has been approved for use by the Food and Drug Administration—is widely used as a vehicle for delivery of pharmaceutically relevant payloads (Danhier et al., 2012; Fernando et al., 2018). Furthermore, the amino groups of PLGA can bind covalently to the carboxyl groups of KGN, thus enhancing the permeability and retention of KGN and promoting its therapeutic effects (Kang et al., 2014; Fan et al., 2018; Chen et al., 2020).

Another critical component of cell-free tissue engineering comprises the biomaterial scaffold. Decellularized extracellular matrix (ECM) derived from cartilage (CECM) can preserve natural ECM components and avoid immune rejection. Therefore, it has been used as a scaffold for cartilage regeneration (Sutherland et al., 2015; Monibi and Cook, 2017). CECM scaffold can provide a suitable microenvironment for the cells it contains; moreover, it exhibits natural adhesion to the cells and growth factors. Notably, CECM is biodegradable, which allows the scaffold to participate in the construction of new cartilage tissue. All of these characteristics promote cell proliferation and functional expression (Yang et al., 2008; Ravindran et al., 2015; Oh et al., 2018; Wang et al., 2018). However, its rapid degradation rate and weak mechanical performance limit the application of ECM scaffold in tissue engineering (Oh et al., 2018). Recently, the introduction of composite scaffolds based on ECM and polymers has provided mechanically and chemically stable properties, while retaining biocompatibility and biodegradability; this offers a new strategy for application of the CECM scaffold. In our previous study, we designed a three-layer biomimetic porous scaffold based on CECM, nano-hydroxyapatite, and silk fibroin. PLGA microspheres encapsulating growth factor rhBMP-2 and TGF-β3 were loaded into the three-layer scaffold to form a slow-release biomimetic osteochondral scaffold. This composite scaffold was able to effectively repair osteochondral defects (Dong et al., 2020).

Here, we fabricated cartilage-derived ECM scaffolds that contained PLGA microspheres or nanoparticles for use in controlled delivery of KGN; the constructs were designed to promote cartilage repair by means of simple procedures. Without the requirement for cell transplantation, this system could recruit endogenous BMSCs from the bone marrow cavity through microfracture apertures. This study was performed to: (1) characterize the ability of the PLGA(KGN)/CECM composite scaffold to support sustained release and chondrogenic differentiation ability *in vitro*, and (2) to evaluate the effects of implantation of PLGA(KGN)/CECM composite scaffold on articular cartilage defects *in vivo*.

## Materials and Methods

### Materials and Reagents

CECM was obtained from fresh porcine articular cartilage by decellularization, in accordance with methods described previously (Yang et al., 2008). Other materials, including biochemical and chemical reagents, are listed in the supporting information.

### Preparation of KGN-Encapsulating PLGA Microspheres and Nanoparticles

PLGA microspheres encapsulating KGN were prepared by the solid-oil-water double solvent evaporation technique. Briefly, PLGA (MedChemexpress, NJ, United States) was dissolved in dichloromethane to approach a final mass fraction of 5%. KGN was dissolved in dimethyl sulfoxide and then mixed with 5% PLGA. The mixture was then added dropwise into 1% polyvinyl alcohol (PVA) solution at an oil phase:water phase volume ratio of 1:20 and emulsified at 16,000 rpm for 3 min, using a high-shear homogenizer in an ice bath. The suspension was then transferred to a magnetic stirrer and stirred overnight at room temperature. Finally, the suspension was centrifuged and the sediments were washed with deionizer water several times. KGN-PLGA microspheres were obtained after the sediments had been freeze-dried for 24 h and stored at −20°C. PLGA nanospheres were prepared by ultrasonication. First, 2.5% PLGA solution containing KGN was added in a dropwise manner to 2.5% PVA solution at an oil phase: water phase volume ratio of 1:5; it was then emulsified by ultrasonication (500 W, 3 min) to prepare an oil/water emulsion. Subsequently, the ethyl acetate was extracted by adding six volumes of 0.5% PVA aqueous solution to the emulsion. Subsequent steps were performed as described for the preparation of PLGA microspheres.

### Morphology Observations and Size Distribution Measurements of Microspheres and Nanoparticles

The freeze-dried PLGA microspheres Were uniformly coated on the surface of the conductive adhesive of the sample table. The shape and surface morphology of PLGA microspheres Were observed by scanning electron microscope (SEM; Quanta 200; FEI, Hillsboro, OR, United States). To determine the morphology of the PLGA nanoparticles, the nanoparticles Were resuspended by deionized water and dripped on a copper mesh and then visualized by transmission electron microscopy (TEM; HT7700 Exalens, Hitachi, Japan). The sizes of the microspheres and nanoparticles Were measured by Zetasizer Nano ZS90 (Malvern Instruments, Worcestershire, United Kingdom).

### Extraction of Cartilage-Derived ECM

The cartilage used in the ECM-derived scaffold was separated with a scalpel from the femoral condyle and patellar groove. The cartilage was cut into small slices (1 mm^3^) using eye scissors, then washed several times with phosphate-buffered saline (PBS). The cleaned cartilage pieces were then placed in an appropriate amount of Tris–HCl; they were disrupted and decellularized using a tissue homogenizer to create a cartilage slurry. The homogenized tissue was centrifuged at 4°C and the supernatant was removed. Next, the sediments were incubated with 1% Triton X-100 in hypotonic Tris–HCl (Solarbio, Beijing, China) with gentle agitation at 4°C for 24 h; they were rinsed several times with deionizer water, then incubated for 12 h in DNase (Sigma-Aldrich, NJ, United States) and RNase (Sigma-Aldrich, NJ, United States) with agitation at 37°C. Finally, the homogenate was centrifuged and washed as described above. The resulting sediment was freeze-dried and stored at −20°C for further use.

### Fabrication of KGN-PLGA Microspheres and CECM Scaffold Composite System

Freeze-dried CECM was resuspended in deionizer water to form a 3% (w/v) suspension. An adequate amount of KGN (MedChemexpress, Rocky Hill, NJ, United States) was then added to the suspension. The mixture was poured into a cylindrical mould, frozen at −80°C for 72 h, and freeze-dried for 48 h. The resulting scaffolds were sterilized and trimmed to an appropriate size. Next, cross-linking was performed in anhydrous ethanol solution containing 50 mM 1-ethyl-3-(3-dimethylaminopropyl) carbodiimide (EDC) and 20 mM *N*-hydroxysuccinimide (NHS) for 24 h at 4°C; cross-linked scaffolds were rinsed several times with PBS. The process produced PLGA microsphere-embedded CECM composite scaffolds with diameter of approximately 5 mm and depth of approximately 3 mm. Scaffolds loaded with TGF-β3 were obtained by immersion in 400 ng/mL TGF-β3 (Peprotech, Rocky Hill, NJ, United States) solution (24 h at 4°C) and then freeze-dried.

### Characterization of Scaffolds

The morphology and structure of scaffolds were characterized by scanning SEM. Briefly, the scaffold specimens were cut into thin sections and fixed on the platform. After they had been sputter-coated with gold, all sections were examined by SEM. Pore diameter distributions were determined using ImageJ software (NIH, Bethesda, MD, United States).

### Determination of Encapsulation Efficiency and Drug Loading of Microspheres

First, several concentrations of KGN standard solutions were prepared and the absorption peak area was determined by high-performance liquid chromatography (HPLC). A standard curve was obtained with the concentration as the x-axis and the peak area as the y-axis. Subsequently, the KGN-PLGA microspheres were submerged in 0.1 M NaOH solution at 1 mg/mL and shaken gently at 37°C for 24 h. The peak area was determined by HPLC and the KGN content in the sample (W1) was calculated by the standard curve formula. The encapsulation rate and drug loading amount of KGN in PLGA microspheres were calculated using the following formulae:

$$\mathrm{Encapsulation}\,\mathrm{efficiency}={\text{W}}_{1}/{\text{W}}_{\text{k}}\times 100\%$$

and

$$\mathrm{Drug}\,\mathrm{loading}={\text{W}}_{1}/\left(\text{Wp}+{\text{W}}_{1}\right)\times 100\%,$$

where W_k_ represents the total volume of KGN and Wp represents the total volume of PLGA.

### KGN Release From Scaffolds

First, the two scaffolds (KGN-CECM and PLGA(KGN)-CECM) were soaked in 5 mL of sterile PBS and shaken at 37°C. PBS was collected daily and replaced with 5 mL of fresh PBS. The concentration of KGN in the collected PBS was determined by HPLC, as described above. Thus, sustained-release curves of KGN-CECM and PLGA(KGN)/CECM were obtained.

### Isolation and Culture of Bone Marrow Mesenchymal Stem Cells

BMSCs were isolated from 4-month-old New Zealand White rabbits. All experimental protocols were approved by the Animal Experimental Ethics Committee of Tianjin Hospital. Following injection of 30% urethane (2.2 mL/kg) into the ear vein, BMSCs were obtained from the rabbits by bone marrow aspiration. After the operative region had been shaved and disinfected, a 2-mL aliquot of bone marrow was aspirated from the femur of each rabbit and placed in a sterile centrifuge tube. Following the addition of 5 volumes of Hanks balanced salt solution, the tubes were centrifuged for 7 min at 1,000 rpm; the mixtures were then divided into three layers. The top two layers were transferred to a new tube and resuspended in 5 mL of Dulbecco’s Modified Eagle Medium supplemented with penicillin and 10% fetal bovine serum (Gibco, Grand Island, NY, United States). BMSCs were then cultured at 5 × 106 cells/cm^2^ in 75-mL culture flasks under an atmosphere of 5% CO_2_ at 37°C. Half of the volume of culture medium was changed after 3 days; the entire volume of culture medium was changed within 1 week. Third-generation (P3) cells were stored for further use.

### Chondrogenic Induction *in vitro*

First, four scaffolds were prepared: blank CECM, CECM loaded with PLGA(KGN), CECM loaded with TGF-β3, and CECM loaded with PLGA(KGN) and TGF-β3. BMSCs were suspended at 1 × 10^7^ cells/mL, then dropped into each of the scaffolds to construct cell–scaffold complexes. The cell–scaffold complexes were then incubated in chondrogenic induction media (α-Minimum Essential Medium supplemented with 10% fetal bovine serum, 100 nM dexamethasone, 50 μg/mL vitamin C, 1% ITS, 100 μg/mL sodium pyruvate, 40 μg/mL proline, 30 μg/mL L-glutamate, and 1% antibiotic-antimycotic) for 14 or 28 days under an atmosphere of 5% CO_2_ at 37°C. The cell–scaffold complexes were fixed with 4% paraformaldehyde; dehydrated and embedded in paraffin wax using a tissue dehydrator; processed for histological analysis; and stained with haematoxylin and eosin (H&E), safranin O, and toluidine blue. All procedures were carried out in accordance with the instructions provided by the reagent manufacturers. For quantitative evaluation of aggrecan and type II collagen in the ECM, the cell–scaffold complexes were removed from the incubator, transferred to liquid nitrogen for several minutes, and then stored at −80°C. Aggrecan and type II collagen enzyme-linked immunosorbent assay (ELISA) kits (R&D Systems, Minneapolis, MN, United States) were used in accordance with the manufacturer’s instructions.

### Cytotoxicity Assay

The cytotoxicity of the scaffold resulting from the residual reagents and/or processing was determined by assessment of cell proliferation using cell counting kit-8 (CCK-8; Dojindo, Rockville, MD, United States). Briefly, the four freeze-dried scaffolds were placed in 24-well plates and pre-wetted with culture solution in an incubator for at least 12 h before cell seeding. Then, P3 BMSCs (1 × 107 cells/mL) were seeded onto each scaffold and incubated at 37°C under an atmosphere of 5% CO2 for 7 days. CCK-8 solution was added to each well at 1, 3, 5, and 7 days, in accordance with the manufacturer’s instructions. The absorbances at 450 nm (A450) of the samples were recorded using a microplate reader. Six replicates of each scaffold type were studied.

### Cell Adhesion and Viability Assessment

Cell adhesion and morphology of BMSCs grown on PLGA(KGN)/CECM scaffold were assessed by SEM after 48 h of cell culture. The viability of adhesive cells on the scaffold was measured using a live/dead cell viability assay kit at 1 and 3 days, in accordance with the manufacturer’s instructions. Briefly, the cell–scaffold samples were incubated in a solution containing calcein (2 × 106 M) and ethidium homodimer-1 (4 × 106 M) for 15–20 min at 37°C, under an atmosphere of 5% CO2; they were then rinsed two to three times with sterile PBS. Cell viability was visualized using laser confocal microscopy (Leica). The numbers of live (green fluorescence) and dead (red fluorescence) cells were calculated using ImageJ.

### Chondrogenic Induction *in vivo*

A full-layer cartilage defect model was used to evaluate the ability of the PLGA(KGN)/CECM scaffold to promote chondrogenic induction. This study was approved by the Institutional Research Ethics Committee of Tianjin Medical University. Twenty-four female New Zealand White rabbits (4–5 months old, 2.5–3.0 kg) were randomly divided into four groups and acclimatized for 1 week at Tianjin Institute of Orthopedics and Sports Medicine before the operation. Rabbits were injected with 30% urethane (2.2 mL/kg) through the ear vein. A lateral para-patellar skin incision, approximately 3 cm in length, was made in both knee joints of each rabbit. Following layer-by-layer incision, the joint capsule was opened. The femoral condyle was exposed following patellar dislocation. A corneal trephine was used to drill a full-thickness cartilage defect (diameter of 4 mm and depth of 1 mm). An acne needle was used to prepare a microfracture on the subchondral bone layer. Defect sites were treated with one of the four scaffolds: (1) CECM, (2) CECM-TGFβ3, (3) PLGA(KGN)/CECM, and (4) PLGA(KGN)/CECM-TGFβ3. The incisions were then sutured in a layer-by-layer manner. Each rabbit was intramuscularly injected with 800,000 units of penicillin once per day for 1 week postoperatively. At 4 and 12 weeks postoperatively, all rabbits were sacrificed by anesthesia overdose and whole knees were collected. Finally, the effects on chondrogenic induction *in vivo* and cartilage defect repair were evaluated by general observation, H&E staining, toluidine blue staining, safranin O staining (Solarbio, Beijing, China), and type II collagen and aggrecan Western blotting.

### Statistical Analysis

Data analysis was performed with IBM SPSS Statistics, version 19.0 (IBM Corp., Armonk, NY, United States). Data are presented as the means ± standard deviations. One-way analysis of variance was performed to determine statistical significance, followed by Tukey’s *post hoc* test or Dunnett’s T3 *post hoc* test. For all analyses, *P* < 0.05 was considered to indicate statistical significance.

## Results

### Characterization of PLGA(KGN) Microspheres and Microsphere-Embedded CECM-Derived Scaffolds

**FIGURE 1:**
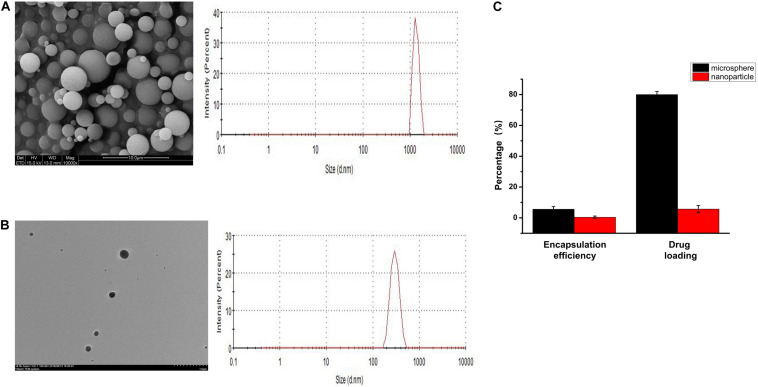
**(A)** SEM image and particle size distribution of PLGA microspheres. **(B)** TEM image and particle size distribution of PLGA nanoparticles. **(C)** Drug loading and encapsulating efficiency rates of PLGA microspheres and PLGA nanoparticles.

**FIGURE 2:**
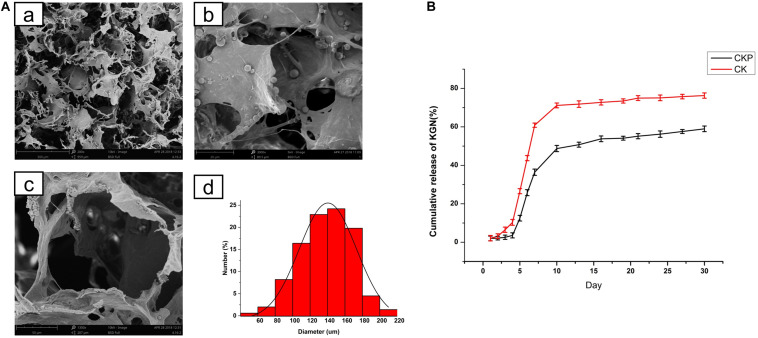
**(A)** Morphology of PLGA(KGN)/CECM composite scaffold: **(a)** PLGA microsphere embedded in CECM scaffold; **(b)** low power (280×); **(c)** pore morphology (1,300×); **(d)** pore size distribution. **(B)** Comparison of release patterns between CECM/PLGA and CECM scaffolds (CKP: PLGA(KGN)/CECM; CK: CECM-KGN).

The prepared PLGA microspheres and nanoparticles loaded with KGN showed a spherical morphology with a smooth external surface; incorporation of KGN did not produce any meaningful changes in the PLGA scaffold structure (Figures 1A,B). NanoZS90 analysis indicated that the mean diameters of PLGA microspheres and nanoparticles were 2.186 μm and 288.8 nm, respectively. A standard curve was constructed to determine KGN concentration. The trapping efficiency and drug loading rate of PLGA microspheres were 80 and 5.6%, respectively; for PLGA nanoparticles, those parameters were 5.7 and 0.4%, respectively (Figure 1C). Therefore, PLGA microspheres were selected for subsequent experiments. The morphology of the PLGA(KGN)/CECM composite scaffold is presented in Figure 2A. The scaffold demonstrated alveolar construction; the interconnected pores of the scaffold exhibited a uniform distribution and the apertures were generally uniform in size. Quantitative analysis revealed that the mean pore diameter was 140 μm (Figure 2A), which met the requirements for use in cartilage tissue engineering. This scaffold was presumed to provide sufficient space for cell adhesion and proliferation, and was considered suitable for the transport of nutritive materials and metabolic waste.

### *In vitro* Release of KGN From Microspheres and Scaffold System

The release of KGN from the CECM/PLGA system (CKP) was monitored for 30 days and compared with the direct release of KGN from the CECM (CK) scaffold. The cumulative release curve (Figure 2B) clearly showed two distinct release patterns of CECM/PLGA and CECM. The release curve of CK reached a plateau after initial rapid release in the first 7 days; cumulative release during that period reached 70% of the gross release. The release curve of CKP demonstrated that there was almost no KGN release in the first 5 days and cumulative release reached approximately 45% of the gross release during the first 10 days; thereafter, it entered a sustained slow-release phase cumulative release reached approximately 45% of the gross release during the first 10 days. The results showed that the CKP displayed controlled and sustained release behavior. Therefore, it was considered suitable for long-term treatment in promoting cartilage regeneration.

### Cell Viability and Proliferation Inside the CECM/PLGA Composite Scaffold

**FIGURE 3:**
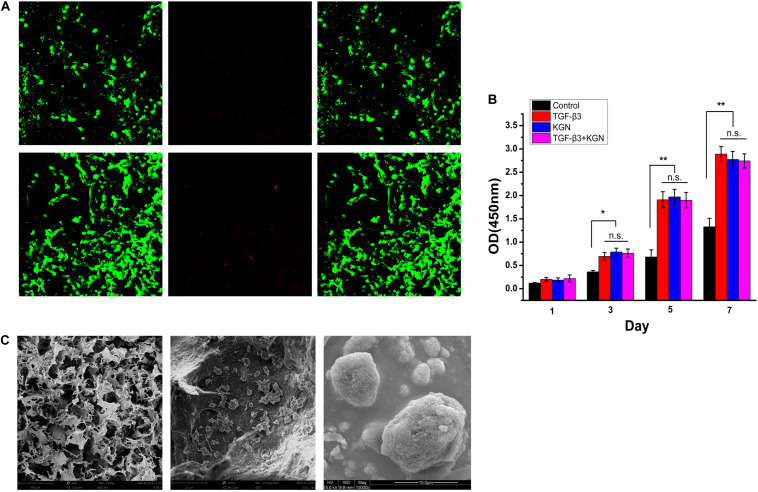
**(A)** Live (green)/dead (red) assessment of BMSCs on composite scaffold. **(B)** Scaffold cytotoxicity assessment using a CCK-8 kit to assess cell proliferation (*P < 0.05; **P < 0.01; n.s., not significant). **(C)** SEM images of BMSC adherence.

To assess the biocompatibility of PLGA(KGN)/CECM scaffolds, BMSCs were seeded in the scaffolds. The biocompatibility of scaffolds was evaluated by cell viability, cell proliferation, and cell adhesion assays. Cell viability was assessed by live/dead staining. As shown in Figure 3A, there were few dead cells on the scaffold, indicating that BMSCs on the scaffold exhibited good cell viability. Cell proliferation, as determined by CCK-8 assay, is shown in Figure 3B. The number of cells increased over time in each group; the three induced groups showed higher rates of increase than the control group, indicating that the scaffold remained safe and non-toxic after embedding of KGN-encapsulated PLGA microspheres. SEM images of cell adhesion in the PLGA(KGN)/CECM composite scaffold are shown in Figure 3C. BMSCs with rough morphology were attached to the inner wall surface of the scaffold; some extended pseudopods and wound through the scaffold. These results further validated that the PLGA(KGN)/CECM scaffold promoted cell adhesion, which could be beneficial for use in cartilage regeneration after *in vivo* implantation.

### Isolation and Chondrogenic Differentiation of the BMSCs

**FIGURE 4:**
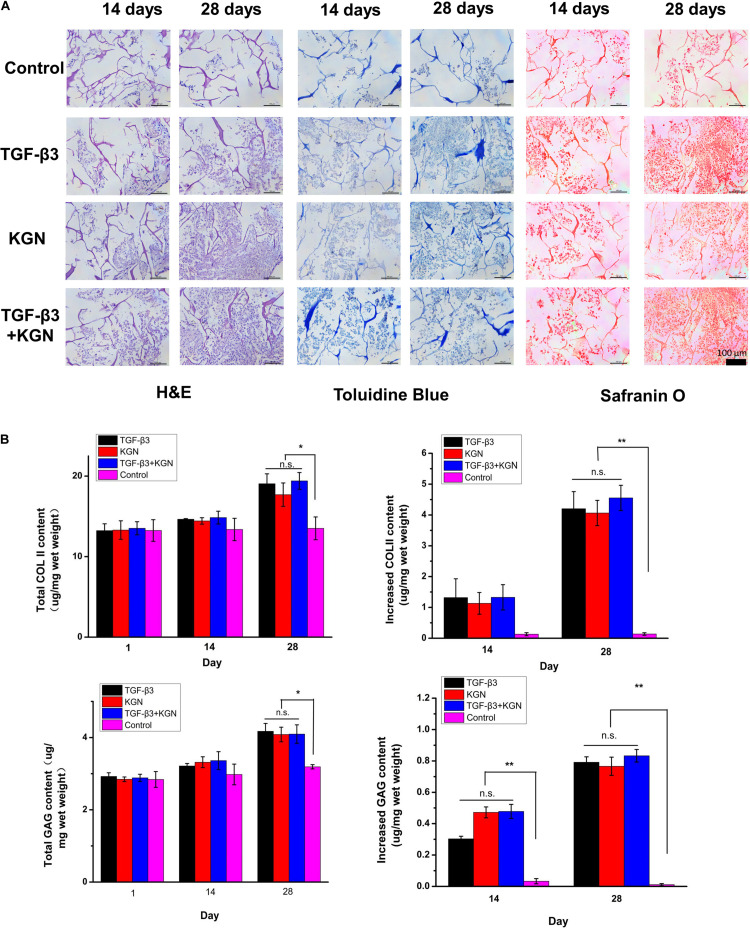
**(A)** Histology of chondrogenic matrix formation within the composite scaffold. **(B)** Quantitative analysis of GAG and type II collagen by ELISA. *p < 0.05, **p < 0.01.

BMSCs were successfully extracted and cultured to passage 3. Following chondrogenesis culture of BMSCs within the four scaffolds for 4 weeks, histological staining (H&E, safranin O, and toluidine blue) and quantitative ELISA analysis of glycosaminoglycan (GAG) and type II collagen were used to compare the chondrogenesis capability of BMSCs among the four scaffolds. As shown in Figure 4A, H&E, toluidine blue, and safranin O staining results indicated that KGN and TGF-β3 both promoted proliferation and chondrogenic differentiation of BMSCs; the cell–scaffold complex exhibited consistent histological findings among the three staining methods. With the exception of the control group, which showed no intact cells or nuclei within CECM scaffolds, the numbers of cells in the other three induced groups (TGF-β3, KGN, and TGF-β3 + KGN) significantly increased over time. The nucleus and cytoplasm were visible by H&E staining, indicating that the cells were in good overall condition; the cell morphology changed from long fusiform to round or oval, indicating that the MSCs had differentiated into chondrocytes. Toluidine blue and safranin O staining revealed that most cells and their secretions in the three induced groups were stained, indicating the presence of type II collagen and GAG components after 28 days of culture.

Longitudinal comparison of the staining conditions of the four groups after 14 and 28 days of culture indicated no significant differences among the TGF-β3, KGN, and TGF-β3 + KGN groups. We presumed that KGN and TGF-β3 did not exhibit a synergistic effect with respect to inducing BMSC differentiation into chondrocytes. These findings were consistent with the results of quantitative analysis of two representative components of hyaline cartilage: GAG and type II collagen (Figure 4B). After 14 days of culture, the total amounts of type II collagen and GAG in the three induced groups did not significantly differ from the total amounts in the control group. After 28 days of culture, the total amounts of type II collagen and GAG were significantly higher in the three induced groups (TGF-β3, KGN, TGF-β3 + KGN) than the control group; however, the total amounts did not significantly differ among the three induced groups. After 14 days of culture, there were no significant differences in the enhancement of type II collagen level among the four groups, while the enhancement of GAG level in the three induced groups significantly differed from the enhancement in the control group. After 28 days of culture, the enhancement of type II collagen level in the three induced groups also significantly differed from the enhancement in the control group.

### Cartilage Repair Assessment in the Rabbit Model

A cartilage defect model was successfully established, as described in the Methods section. At predetermined time points of 8 and 12 weeks, the rabbits were sacrificed and their femoral condyles were harvested. Subsequent assessments were performed to estimate the cartilage regeneration efficiency *in vivo*.

### Macroscopic Observation

**FIGURE 5:**
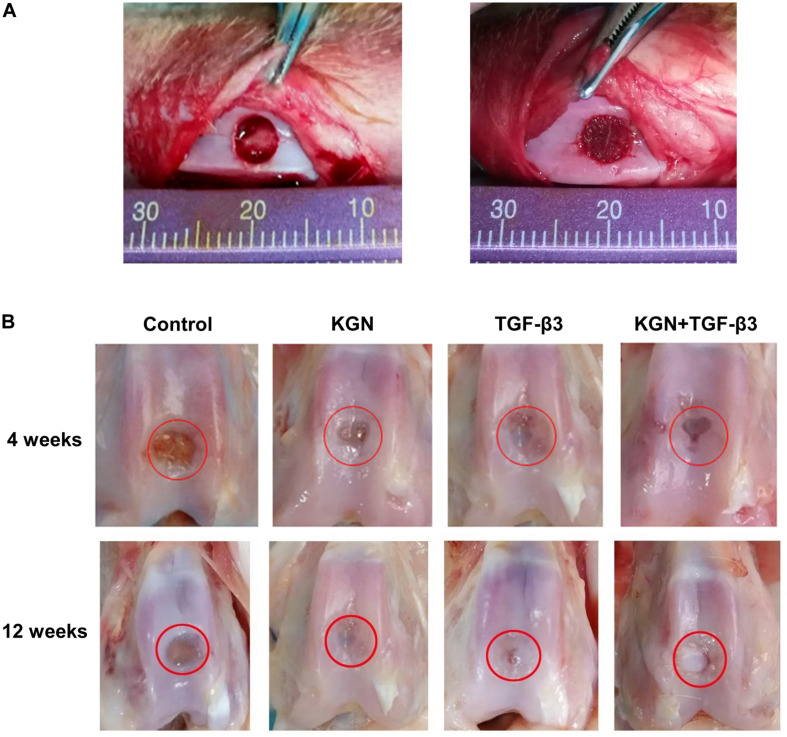
**(A)** Full-thickness cartilage defect model of rabbit femoral trochlea. **(B)** Gross observation at different time points.

Macroscopic observation of repaired cartilage was performed, as shown in Figure 5. At 4 weeks postoperatively, the vestiges of the articular cartilage defect remained, but new tissue began to appear in the three induction groups, whereas it did not in the blank scaffold group (treatment with CECM scaffold alone). At 12 weeks postoperatively, the control group exhibited broad areas of cartilage destruction and surface denudation, while constructs induced with either KGN or TGF-β3 generally showed an intact surface. The boundary with surrounding natural cartilage was less noticeable in the KGN-induced group, compared to the TGF-β3-induced group. Combined induction with both factors did not enhance the repair effect, compared to either KGN or TGF-β3 alone.

### Histological Staining Analysis

**FIGURE 6:**
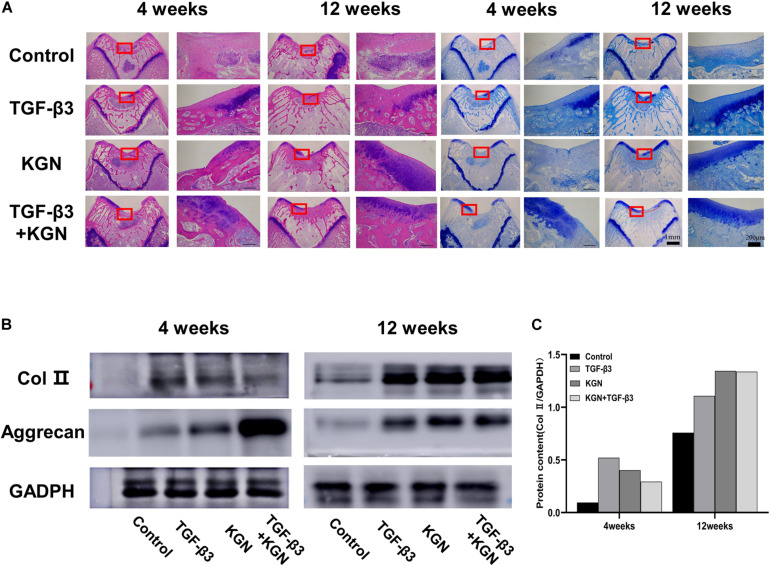
**(A)** Histology of defects at 4 and 12 weeks. **(B)** Western blotting analysis of type II collagen and aggrecan at 4 and 12 weeks. **(C)** Gray value analysis of type II collagen bands.

H&E staining of regenerated cartilage in the four groups is depicted in Figure 6A. At 12 weeks postoperatively, H&E staining revealed cartilage defects in the blank scaffold group. In contrast, cartilage defect areas in the KGN, TGF-β3. and TGF-β3 + KGN groups were mostly covered with new cartilage tissue; the thickness of the new tissue was similar to that of the surrounding normal cartilage layer. The chondrocyte concentration and ECM deposition were significantly enhanced, while the number of inflammatory cells was reduced, compared with 4 weeks postoperatively. Blue dye was evident in toluidine blue staining assays (Figure 6A), further confirming the changes in cartilage matrix content. Compared with the other two groups, staining intensity was higher in the KGN and TGF-β3 + KGN groups that contained higher glucosamine polysaccharide content in the cartilage ECM.

### Western Blotting Analysis

Type II collagen and aggrecan protein expression were further assessed by Western blotting analysis (Figures 6B,C). At both 4 and 8 weeks postoperatively, the three induced groups displayed much stronger bands of type II collagen and aggrecan, compared with the blank scaffold group. However, there were no visible differences among the three induced groups.

## Discussion

Tissue engineering offers novel and effective approaches for repairing articular cartilage defects. While numerous cell-based techniques have been applied to repair and regenerate cartilage defects, these techniques have multiple limitations, including cellular apoptosis and increased risk of disease transmission, resulting in low repair efficiency (Trounson and McDonald, 2015; Huang et al., 2016). Cell-free strategies have been proposed to repair and regenerate the injured cartilage by recruitment of endogenous stem cells, thus avoiding many of the limitations and pitfalls of cell-based strategies and allowing broader clinical application. The present study demonstrated that a cell-free scaffold based on CECM and PLGA microspheres loaded with KGN provided an optimal *in vitro* environment for the attachment and chondrogenesis of BMSCs. Following implantation into a cartilage defect in the rabbit knee joint, the scaffold supported chondrogenic differentiation of endogenous BMSCs, thus promoting the regeneration of hyaline cartilage.

The fabrication of biomimetic scaffolds to recreate the structural features of native articular cartilage is crucial for successful cartilage regeneration. In our previous studies, we developed and characterized a structurally biomimetic oriented scaffold derived from CECM, which could closely mimic the natural chondrogenesis microenvironment (Yang et al., 2008, 2017). The CECM scaffold preserves multiple structural and functional proteins (e.g., fibronectin, hyaluronic acid, and GAG), which could modify the secretion of GAG and type II collagen to further induce specific cellular responses and direct new tissue formation (Sutherland et al., 2015; Ng et al., 2016). In the present study, we combined PLGA and CECM to produce composite scaffolds, which revealed the complementary benefits of the two materials. Notably, the presence of CECM provided good scaffold biocompatibility; additionally, the presence of PLGA conferred adequate mechanical support and relatively long degradation time, thereby matching the neocartilage formation rate (Lih et al., 2016). We showed that the composite scaffold with appropriate pore structure and pore diameter served as a guide for BMSC attachment, distribution, and proliferation; thus facilitating chondrogenic differentiation (Figure 2A). These findings indicated that we had fabricated the physiological structure of native cartilage.

The PLGA microsphere-embedded CECM scaffold can serve to mimic some features of the native ECM; it also enables sustained and localized delivery of bioactive molecules to achieve prolonged exposure of BMSCs to bioactive molecules. Previous studies have shown that the delivery effect of PLGA is related to multiple factors (Wu et al., 2013; Zhu et al., 2019) including the grain size of PLGA spheres; this parameter mainly affects the specific surface area, distribution of hydrophobic drugs in spheres, and polymer degradation rate (Chen et al., 2015; Almeida et al., 2020). Therefore, it is necessary to identify a suitable particle size range that can preserve resources and effectively control KGN release. In the present study, we fabricated PLGA microspheres and nanoparticles for encapsulation of KGN. Following multiple analyses, we chose PLGA microspheres for subsequent experiments because of their considerable advantages in terms of drug loading and encapsulating efficiency (Figure 1C). To evaluate the controlled release effect of the composite scaffold, we monitored the release of KGN for 30 days. The release curve of KGN loaded in the CKP group demonstrated that there was almost no KGN release in the first 5 days and cumulative release reached approximately 45% of the gross release during the first 10 days; thereafter, it entered a sustained slow-release phase (Figure 2B). We speculate that this is due to the protection of CECM scaffolds on PLGA microspheres, which prevents the degradation of PLGA microspheres caused by rapid water entry. Therefore, in the first 5 days, only the drugs on the surface of the microspheres were released. The microspheres began to degrade in 5–10 days, which accelerated the drug release inside the microspheres. The CKP group demonstrated more robust sustained-release capability, compared to the CK group with more obvious sudden release; this implied that the composite scaffold had a double-sustained effect on KGN release, such that it may be suitable for long-acting administration of KGN in clinical applications. Notably, the PLGA microsphere-embedded scaffolds improved the diffusion path because KGN was confined to the core region of the PLGA microspheres and initially dispersed through the shell polymer before departure from the CECM. In addition, the amine groups of PLGA and the carboxyl groups of KGN exhibited strong covalent linkages, which aided in controlled release.

The bioactivity of released KGN was assessed by measuring the ability of the KGN-loaded scaffold to promote BMSC growth and chondrogenesis. Our assessment indicated that this KGN-releasing system promoted BMSC viability and proliferation (Figure 3); it also enhanced proteoglycan deposition and type II collagen content compared to the control group, potentially promoting the regeneration of cartilage *in vitro* (Figure 4). These findings were consistent with previous research demonstrating the chondrogenic effect of KGN supplementation on BMSC growth (Johnson et al., 2012). Furthermore, *in vivo* evidence indicated nearly complete repair of cartilage defects at 12 weeks after implantation of the KGN-releasing composite scaffold. Notably, the neocartilage was integrated with its surrounding tissue and subchondral bone (Figure 6A). The levels of type II collagen and GAG expression were elevated in the three induced groups, compared to the control group (Figures 6B,C), thereby improving the quantity and quality of regenerated tissue at the chondral interface. A previous study demonstrated similar improvements in proliferation and chondrogenesis of human adipose-derived MSCs or synovium-derived MSCs by treatment with KGN (Shi et al., 2016; Zhu et al., 2017). Therefore, we speculated that the cartilage repair process could be continued by recruiting other sources of MSCs when BMSCs were no longer enriched after microfracture healing. Further studies are required to examine this aspect of cartilage repair treatment.

In the present study, KGN loaded in CECM/PLGA was able to induce differentiation of BMSCs in a manner similar to that of TGF-β3 loaded in CECM, but combined treatment did not show synergistic effects. Our observations were consistent with the findings by Zhu et al., who demonstrated that KGN was a suitable replacement for TGF-β3 in terms of type II collagen induction and aggrecan deposition, but that it did not promote or enhance the effects of TGF-β3 (Zhu et al., 2017). However, Jia et al. reported that the combination of TGF-β3 and KGN synergistically promoted chondrogenic differentiation of synovial fluid-derived MSCs via the Smad 2/3 pathway (Jia et al., 2019). Recently, Jing and colleagues also reported that KGN preconditioning could exert dually beneficial effects on TGF-β3-induced chondrogenic differentiation of human umbilical cord MSCs (Jing et al., 2019). Therefore, we speculate that the synergistic effect of combined KGN and TGF-β3 treatment may be based on sequential application consisting of KGN preconditioning with subsequent TGF-β3 induction; the synergism disappeared when the sequential application was disrupted in this study. Another possibility is that the synergistic effect may only be observed for some specific type(s) of MSCs (e.g., adipose-derived MSCs). Further studies are required to explore the mechanisms underlying these effects.

To sum up, although the CECM scaffolds and PLGA microspheres have been widely performed in the cartilage tissue engineering, the combined application of these two materials is rarely investigated. In this study, we updated the application approach of KGN through loading KGN into the CECM/PLGA scaffold. The porous CECM/PLGA scaffolds can not only cover the advantages of CECM porous scaffolds and PLGA microspheres, but also sustain the KGN release. Besides, the scaffold in the present study showed the similar cartilage regeneration efficiency with TGF-β3, hence, we hope that KGN can be a suitable replacement for TGF-β3 in cartilage tissue engineering to avoid some adverse effects caused by excessive TGF-β3 (Mi et al., 2003).

This study had some limitations. First, the biomechanical properties of the scaffold were not fully analyzed. Second, the present results were obtained in a rabbit model. Therefore, before human clinical trials, the scaffold should be examined in large-animal models that are immunologically similar to humans, which can provide evidence of its safety and efficiency.

## Conclusion

We successfully prepared a composite scaffold by blending KGN-encapsulated PLGA microspheres and CECM. *In vitro* assessments demonstrated that the PLGA(KGN)/CECM composite scaffold prolonged the activity of KGN and supported the attachment, proliferation, and differentiation of BMSCs. Initial *in vivo* analysis indicated that the PLGA(KGN)/CECM induced superior hyaline-like neocartilage repair; the neocartilage successfully integrated with its surrounding tissue in a rabbit model. Moreover, the PLGA(KGN)/CECM system avoids difficult cell manipulation during preparation, providing a new small molecule-based strategy for cartilage tissue engineering.

## Data Availability Statement

The original contributions presented in the study are included in the article/supplementary material, further inquiries can be directed to the corresponding author/s.

## Ethics Statement

The animal study was reviewed and approved by Tianjin Medical University.

## Author Contributions

YHZ and YMZ designed and conceptualized the work. XGZ and RZ carried out the study and collected the crucial background information. YH, YL, and XTZ collected the data. MS analyzed and interpreted the data. XGZ and YHZ wrote the manuscript. YX, MW, and YZ revised the manuscript critically. All authors have read and approved the final manuscript.

## Conflict of Interest

The authors declare that the research was conducted in the absence of any commercial or financial relationships that could be construed as a potential conflict of interest.
